# Targets and Effects of Yessotoxin, Okadaic Acid and Palytoxin: A Differential Review

**DOI:** 10.3390/md8030658

**Published:** 2010-03-16

**Authors:** Antonella Franchini, Davide Malagoli, Enzo Ottaviani

**Affiliations:** Department of Biology, University of Modena and Reggio Emilia, 41125 Modena, Italy; E-Mails: antonella.franchini@unimore.it (A.F.); davide.malagoli@unimore.it (D.M.)

**Keywords:** development, FETAX, marine toxins, invertebrate, vertebrate, cell cultures, toxin targets

## Abstract

In this review, we focus on processes, organs and systems targeted by the marine toxins yessotoxin (YTX), okadaic acid (OA) and palytoxin (PTX). The effects of YTX and their basis are analyzed from data collected in the mollusc *Mytilus galloprovincialis*, the annelid *Enchytraeus crypticus*, Swiss CD1 mice and invertebrate and vertebrate cell cultures. OA and PTX, two toxins with a better established mode of action, are analyzed with regard to their effects on development. The amphibian *Xenopus laevis* is used as a model, and the Frog Embryo Teratogenesis Assay-*Xenopus* (FETAX) as the experimental protocol.

## 1. Introduction

The studies and reviews on the marine toxins are becoming more numerous due to the increase in the frequency and the distribution in many regions of the world of algal toxins that can damage public health, fishing, fish and shellfish cultures, and marine ecosystems. In particular, it has been reported that algal toxins are responsible for more than 50,000–500,000 intoxication incidents in humans per year, with an overall mortality rate of 1.5% on a global basis [1]. Furthermore, toxic algae are also responsible for the death of animals dependent on the marine food web [2–5]. The human ingestion of contaminated marine organisms provokes pathological symptoms that were collectively labeled more than 40 years ago with the term biopoisonings [6]. Among the approximately 5,000 marine algal species, less than 100, mainly from the dinoflagellates and diatoms taxa, produce toxins [7]. The increasing presence of algal toxins determined by various known (water ballast from merchant ships, eutrophication of the coastal areas, anthropogenic eutrophication, *etc.*) and unknown agents has been called “harmful algal bloom” [8,9]. Algal toxins may be classified by function of their target and seafood poisoning syndromes [1].

Here we report the findings from our and other groups on the marine toxins yessotoxin (YTX), okadaic acid (OA) and palytoxin (PTX). However, since toxicological properties of YTX and the molecular targets of OA and PTX have already been exhaustively reviewed [10–14], we will focus our analysis on the possible mechanisms of action and target organs for YTX and will summarize the effects that OA and PTX exert on vertebrate embryos and their development. We describe experiments that have studied YTX effects on mussels, mice and both invertebrate and vertebrate cell cultures, in order to isolate possible biological targets of YTX at both organ and cellular level. For PTX and OA, we report experiments performed either on invertebrates or using the amphibian *Xenopus laevis* as a model and the Frog Embryo Teratogenesis Assay-*Xenopus* (FETAX) as the experimental protocol.

## 2. Yessotoxin (YTX)

### 2.1. General proprieties

YTX is a sulfated polyether compound originally isolated in Japan from the digestive gland of the scallop *Patinopecten yessoensis* [15], and is produced by phytoplanktonic microalgae of some dinoflagellate species. As they were extracted together with toxins provoking clinical symptoms of diarrheic shellfish poisoning (DSP), YTX and its derivatives were originally classified among diarrheic shellfish toxins (DSTs). However, unlike the latter, YTXs do not provoke diarrhea and are not lethal to mice after oral intake even at a dose of 1 mg/kg [16]. Nevertheless, the precise mode of action remains currently unknown, even if YTX seems to be a potent phosphodiesterase activator [17]. The toxicological data from studies carried out in rodents are conflicting, in particular regarding the lethal dose concentration after intraperitoneal injection, which ranged from 80–1,000 μg/kg [16,18–20]. The main internal organs examined after mice death did not show any significant histomorphological changes clearly related to the toxin [16], while the main target organ after acute or daily repeated oral administration has been long reported to be the heart tissue [14,16,18,20]. Despite these somehow discrepant observations, evidence of YTX action on neuronal cells, which are vulnerable biological targets, has also been reported. Death of animals during mouse experiments was preceded by symptoms that included motor discoordination and jumping, while cerebellar cortical alterations were also demonstrated [21,22]. Several i*n vitro* studies have been performed in order to elucidate the mechanism(s) of action and the possible target of YTX at cellular level. These experiments have confirmed the results of morphological analyses and suggest a high cytotoxicity for YTX affecting a variety of cellular activities. YTX has been seen to promote the activity of caspases 3 and 7 in HeLa cells [23], it opens the permeability transition pore in rat liver mitochondria [24] and causes cytoskeletal disruption together with apoptosis in cultured cerebellar neurons [25]. Very recently, the effects of YTX on cytoskeletal components of vertebrate cells has been connected with a reduced phagocytic activity and the inhibition of phagosome maturation in the J774 macrophage cell line [26].

### 2.2. Data derived from experiments on invertebrate and vertebrate cell lines

**Figure 1:**
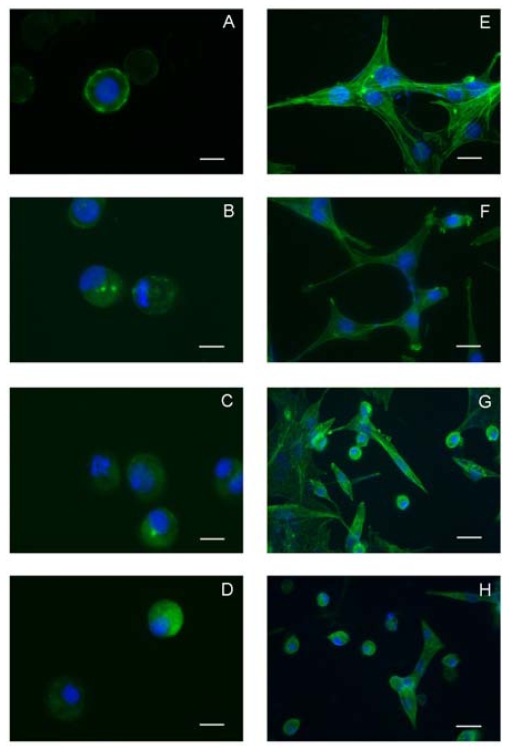
Time-dependent depolymerization of F-actin promoted by YTX in IPLB-LdFB and NIH3T3 cell lines evidenced by FITC-phalloidin labeling and DAPI nuclear counterstaining. IPLB-LdFB: control cells (A); and IPLB-LdFB after 24 h (B), 48 h (C) and 72 h (D) incubation with 100 nM YTX; NIH3T3 control cells (E); and NIH3T3 cells after 24 h (F); 48 h (G); 72 h (H) incubation with 100 nM YTX. Bar = 10 μm. (Reprinted with permission from [30]).

**Figure 2:**
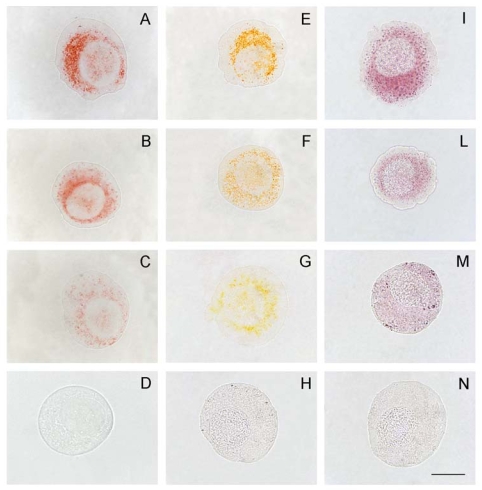
Lysosomal damage promoted by YTX in IPLB-LdFB cell lines evidenced by neutral red (A–D), acridine orange (E–H) and acid phosphatase activity (I–N) methods. A, E, I) IPLB-LdFB control cells; B, F, L) IPLB-LdFB cells after 8 h incubation with 100 nM YTX; C, G, M) IPLB-LdFB cells after 12 h incubation with 100 nM YTX; D, H, N) IPLB-LdFB cells after 24 h incubation with 100 nM YTX. Bar = 10 μm. (Reprinted with permission from [30]).

Although several reports have confirmed the cytotoxic potentialities of YTX, it has remained a highly interesting but scattered scenario, as the primitive target of YTX remains unclear [27]. In this perspective, we performed experiments testing an insect and a mammalian cell line in parallel. The effects of 10 and 100 nM YTX on the IPLB-LdFB insect cell line, derived from the larval fat body of the gypsy moth *Lymantria dispar* [28,29], were compared with those on the mouse fibroblast NIH3T3 cell line [30]. The experiments confirmed that YTX displays a concentration-dependent and cell-specific cytotoxic activity. These findings also demonstrated that in both IPLB-LdFB and NIH3T3 cells, F-actin microfilaments are progressively depolymerized within 48 h after the exposure to YTX (Figure 1). Moreover, by using a combination of morphological markers for acidic compartments, it was possible to demonstrate that in both the cell types, the lysosomal content moved quickly into the cytoplasm, thus indicating that lysosomes may be the first cellular component damaged by YTX (Figure 2) [30]. In our hypothesis, the documented increase in Ca^2+^ concentration that follows YTX exposure in different cell types [14,31,32] could promote the spilling of lysosomal content into the cytoplasm, in turn promoting all the other well established processes, such as actin depolymerization.

### 2.3. Data derived from experiments on the mussel Mytilus galloprovincialis

Algal toxins can be accumulated by filter feeding molluscs, such as mussels. Therefore, an important task for comprehending the possible ecological impact of algal toxins is the assessment of their effects on those marine organisms. Unfortunately, while several articles report on the isolation of YTX from mussels of different origin [33–35], a very limited number of studies is available on the possible effects of YTX on mussel biology. Immunocytochemical experiments have evidenced that the mussel *M. galloprovincialis* accumulates YTX into the cytoplasm of circulating immunocytes (hemocytes endowed with phagocytic activity and several immune-related functions) and the tubules and ducts of the digestive gland [36]. Mussels can accumulate YTX without displaying apparent damage, and it has been suggested that this could be due to metabolic processes that would reduce its toxicity [37]. We have investigated the direct effects of the toxin on mussel immunocytes. Our observations indicate that the toxin alone is not able to influence a fundamental activity of immunocytes, *i.e.*, cell motility. However, when the immunocytes were co-stimulated with bacterial extracts, the effects of the toxin were clearly observable [38]. Further experiments revealed that the effects of YTX on immunocytes is imputable to an augmented concentration of cytosolic Ca^2+^ [32] as has also been suggested for human lymphocytes [31]. Furthermore, the link between YTX and immune functions has also been very recently confirmed in mammalian J774 phagocytes [26]. Intriguingly, the effects of YTX on J774 cells included an increased expression of cytokines [26], suggesting that possible effects of the toxin might include/determine an altered pattern of expression in immune-related genes.

### 2.4. Data derived from experiments on Swiss CD1 mice

In spite of the increasing number of studies from different models focusing on the cellular activities affected by YTX, *in vivo* analysis performed on mice revealed conflicting toxicity data, and the discrepancies have been attributed to experimental laboratory conditions and the use of mice of different age, strain and gender [39]. The histopathological results on the target organs are not exhaustive and, in particular, little attention has been paid to pathological changes induced by algal toxin treatment in some systems *i.e.*, lymphoid tissue.

**Figure 3:**
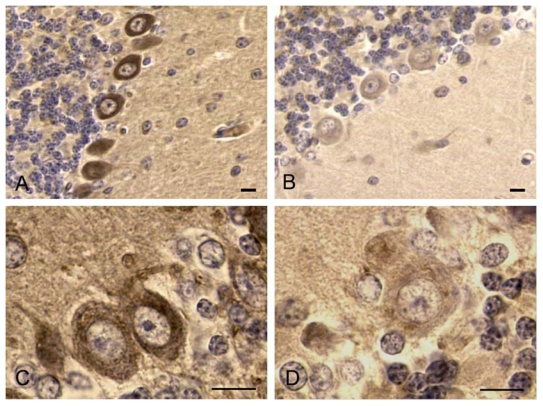
Immunolocalization of Ca^2+^-binding proteins and cytoskeleton components in the cerebellum cortex from control (A, C) and 420 μg/kg YTX injected mice (B, D). In treated samples, the positivity to anti-calbindin D-28K mAb (A, B) and to anti-β-tubulin (C, D) decrease. Bar = 10 μm. (Reprinted with permission, modified from [22]).

The effects of intraperitoneal injection of a lethal dose (420 μg/kg, causing death after less than 2 h) and a 24 h sub-lethal dose (10 μg/kg) of YTX have been studied in the brain, duodenum and thymus of male Swiss CD1 mice [22,40]. The cerebellum was demonstrated to be the most sensitive area of the brain, and severe morpho-functional changes were found in the Purkinje cell layer of the cortex. Indeed, after toxin treatment, these cells were irregularly distributed and showed an altered structure, with Nissl body shrinkage, nuclear chromatin condensation and significant changes in immunoreactivity to calcium-binding proteins and cytoskeletal components. With respect to controls, positivity was found to increase for the S100 protein, to decrease for calbindin D-28K (Figure 3), while the neuronal microtubule and neurofilament pattern organization appeared altered with a decreased immunoreactivity to β-tubulin (Figure 3) and neurofilament antibodies. In contrast, YTX did not induce any significant morpho-functional modifications in other brain regions *i.e.*, cerebral cortex [22,40].

The duodenum was more sensitive to the higher YTX dose. In spite of the preserved general organization, a higher number of blood cells infiltrated between epithelial cells of villi and some blood cells, mainly lymphocytes, present in mucosa connective and epithelial tissue and in lymphoid Payer’s patches displayed apoptotic phenotypes. A larger number of cells, mostly granulocytes and macrophages, in the epithelial layer and underlying connective tissue were strongly immunoreactive for IL-6 and TNF-α while the number of cells positive to the anti-IL-8 antibody decreased. The sub-lethal YTX dose did not induce evident histological changes, and cytokine responses also showed a similar trend [40].

**Figure 4:**
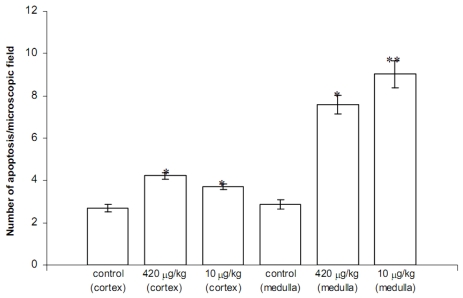
Number of apoptotic cells recorded in thymic sections from control, 420 and 10 μg/kg YTX injected mice (*, ** p < 0.05 *versus* control; ** p < 0.05 *versus* *). (Reprinted with permission from [40]).

In contrast to the duodenum, the thymus reacted to both YTX doses, but the structural damage was more severe at the sub-lethal concentration. After injecting mice with the lethal dose of 420 g/kg YTX, less compact areas with a reduced number of thymocytes were present in thymus cortex. Moreover, apoptotic phenotypes, mostly of thymocytes, increased significantly in the thymic medulla in particular, while mitosis activity was more stimulated in the cortex. The sub-lethal YTX dose induced mainly cortico-medullary junction and medulla responses, with a significantly higher number of apoptotic cells than in control samples or following the lethal treatment (Figure 4).

**Figure 5:**
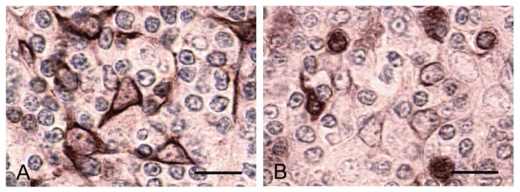
Sections of thymus from control (A) and 10 μg/kg YTX injected mice (B) immunostained with anti-cytokeratin 1/5/10/14 mAb. Note the changes in organization of the medullary epithelial compartment with modifications in cell morphology and cytokeratin immunoreactivity. Bar = 10 μm. (Reprinted with permission, modified from [40]).

The mitosis was comparable to that observed at the higher dose. Round structures resembling Hassall’s corpuscles containing heterogeneous secretory material, cell debris and necrotic nuclei and dendritic cells with cytological features of active phagocytic cells were also observed in medulla. After YTX treatment, the most damaged cell population appeared to be the epithelial cells that were found to loose their reticular organization, to change cell morphology from a stellate to a round form and immunoreactivity to different molecular weight (MW) cytokeratins (Figure 5). The newly formed medullary Hassall’s corpuscle-like bodies were also immunoreactive to higher MW cytokeratins. With regard to cytokine response, changes in comparison to controls were observed with both YTX concentrations. More cells, mostly dendritic cells in cortico-medullary junction and medulla, were immunoreactive to IL-6, while a lower number of IL-1 and IL-8 immunoreactive cells were seen in the cortex [40].

On the whole, our findings suggest that the thymus and, in general, the immune system are the main targets of YTX at both the concentrations used, while alterations to the cerebellum are detected only at the lethal dose. Early indicators of the neurological disorders induced by acute YTX toxicity may be considered modifications of cytoskeletal components and of intracellular Ca^2+^-binding protein levels observed in Purkinje cells. The morpho-functional changes induced in the thymus are compatible with those reported in thymic tumours [41–44] supporting the thesis of possible tumorigenic implications for this toxin.

## 3. Okadaic Acid (OA) and Palytoxin (PTX)

### 3.1. General proprieties

Okadaic acid (OA) is a lipophilic compound produced by several marine dinoflagellates belonging to the genera *Dinophysis* and *Prorocentrum.* It is almost exclusively accumulated in mussel digestive gland and the consumption of these animals provokes a syndrome in humans known as DSP, characterized by severe gastrointestinal symptoms [45,46]. OA acts by inhibiting serine/threonine protein phosphatases (PP1 and specially PP2A, [47]) that results in the hyperphosphorylation of many cell proteins and the dis-regulation of a variety of cellular processes. *In vivo* studies in mice have reported the distribution and excretion of OA following oral administration, as well as morpho-functional changes of organs targeted by the toxin. OA is primarily considered an enterotoxin [16,48–50], and low oral doses are also able to provoke both immunostimulation and systemic immunotoxicity in Swiss CD1 mice [51]. The extent of the OA-induced injuries and the toxin organotrophicity are dose-related [16] and may be determined by the administration route. After intravenous administration, OA acts as a hepatotoxin with undetectable effects on the intestine, but also has an impact on cytoskeletal elements at sub-lethal doses [52]. Moreover, the toxin is known to act as a potent neurotoxin for cultured neurons [53], to induce apoptotic events in various cell lines [54,55] and to exert tumor promoting activity in various organs [56–58].

Palytoxin (PTX) is a large, water soluble polyalcohol first isolated from the soft coral of the genus *Palythoa* [59], subsequently found in a variety of marine organisms ranging from dinoflagellates to fish and implicated in seafood poisoning with potential danger to public health. Toxicological studies have shown its toxicity through different exposure routes and with different lethal exposure concentrations in various animal models, as summarized by Deeds and Schwartz [60]. Various symptoms have been described in relation to the PTX exposure route, and rhabdomyolysis, a syndrome involving skeletal muscle injury provoked by sarcolemma disruption, is a complication of the poisoning [61–63]. Acute oral administration in mice induced histological and ultrastructural changes in several organs, such as liver, pancreas, cardiac and skeletal muscle cells [64]. The toxin is known to affect cellular functions by selective binding to the membrane Na^+^-K^+^ ATPase, which is essential in maintaining ionic gradients, and converting the pump into a non-selective channel [65,66]. The induced ion flux alterations are reported to target pathways involved in cytoskeletal dynamics within different cellular models [12]. Additionally, PTX has been identified as a potent tumor promoter which modulates MAP protein kinase cascades by different mechanisms [11].

### 3.2. Data derived from experiments on invertebrate models

**Figure 6:**
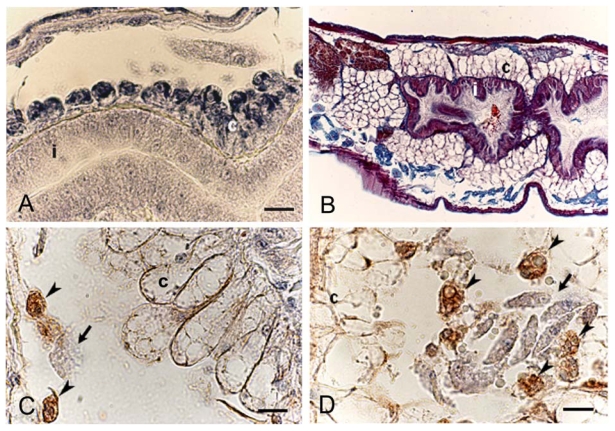
Longitudinal sections from *E. crypticus* controls (A, C) and specimens treated with 100 nM OA for 24 h (D) and 200 nM OA for 12 h (B) stained with gallocyanin-chrome alum (A), Mallory-Azan stain (B) and immunostained with anti-IL-6 polyclonal antibody (C, D). (A) The chloragogenous tissue (c) from controls formed one or two layers of round vacuolated and basophilic cells surrounding the intestine (i). (B) After OA treatment, the tissue showed a higher number of cell layers and expanded into the celomatic cavity. The toxin also induced an increase in the number of circulating celomocytes. (C, D) Immunonegative chloragocytic cells, arrows; immunopositive amoebocytes, arrowheads. Bar = 10 μm. (Reprinted with permission, modified from [68]).

The effects of OA have been studied on the structural organization of the potworm *Enchytraeus crypticus*, an annelid species widely used in ecotoxicological laboratories to test the effects of chemical stress [67]. This experimental model proved very sensitive to OA treatment (added to food) in a time- and dose-related manner [68]. After 48 h of treatment at the lower dose (100 nM), the main organs were not particularly affected, except for a swelling of the celomatic cavity and the number of circulating celomocytes. Conversely, at 400 nM OA, a general cell suffering was observed in the animal’s main organs. After 12 h, and to a greater extent after 48 h, of treatment with an intermediate OA concentration (200 nM), the structural organization of the chloragogenous tissue, a layer of cells that separate the “blood” from the celomic fluid [69], appeared modified (Figure 6), and an immune response involving a higher number of circulating celomocytes immunoreactive to anti-IL-6 antibody was observed (Figure 6).

The nervous system, in particular the ventral nerve cord, was also affected, presenting fewer IL-6 immunoreactive cells [68]. The ability of *E. crypticus* of different ages samples (25 days and three months) to restore the induced toxicological injuries was also analyzed, and the results indicated that the older worms were more sensitive to the toxin, with the capacity to recover lower than that found in specimens aged 25 days. First signs of restoration from the morpho-functional modifications were seen in younger worms at 48 h, with an almost complete recovery within one week. In older animals, the morphology of the chloragogenous tissue was not restored, while a reduction in celomocyte number was found after one week [70].

As mentioned above for YTX, the analysis of biological effects of toxins on those animals that naturally accumulate them [71] still remains a relatively unexplored field. The effects of OA and PTX on the phagocytic activity of mussel immunocytes, have been tested both in normal conditions and after exposure to possible stressful situations [72]. Our data indicate that OA effects strictly depend on the incubation temperature [72]. Indeed, only when the immunocytes were exposed to the toxin at the temperature of 25 °C was it possible to observe a significant increase in the percentage of phagocytizing immunocytes. At the temperature of 20 °C, OA appeared to have no effect on the phagocytic activity of molluscan immunocytes [72]. PTX augmented the percentage of phagocytizing cells independently of the temperature. Unlike YTX and OA, PTX also seems to intervene actively in the signaling pathway of mussel immunocytes, since increased immunoreactivity towards the phosphorylated form of the p38 MAP kinase was observed through immunoblot experiments. Moreover, the effects of PTX on immunocytes were annulled by the p38 MAP kinase inhibitor SB 203580 [72]. Since in *M. galloprovincialis* immunocytes, the signaling pathway involving p38 MAP kinase can be differentially regulated in the presence of mild stress [73], the effects of PTX, which also rely on that pathway, may vary on the basis on mussel conditions. Altogether, the data collected in *M. galloprovincialis* indicate that the neutrality displayed by the accumulated toxins may just be apparent, since toxin effects can change when the biochemical milieu inside the cells changes as a consequence of environmental modifications.

### 3.3. Data derived from experiments on the amphibian X. laevis

FETAX protocol has been widely described and applied to different substances [74–79], and it is a powerful and flexible bioassay for developmental toxicants that makes use of the embryos of the anuran *X. laevis*. In embryos, this biological assay can verify the effects of a toxicant in terms of mortality, delayed growth and embryo malformation. The test has numerous advantages: the use of a non-mammalian species, *X. laevis*, that is easy to maintain and can be bred throughout the year; the assay can be carried out in a short time and with relatively low costs; the method guarantees a good repeatability of results and provides a large amount of data for statistical analysis. The assay has been widely described and applied for various substances [74–79] and has been recently utilized, together with further histological and molecular investigations, to investigate the effects of OA and PTX on vertebrate development [80–82].

**Figure 7:**
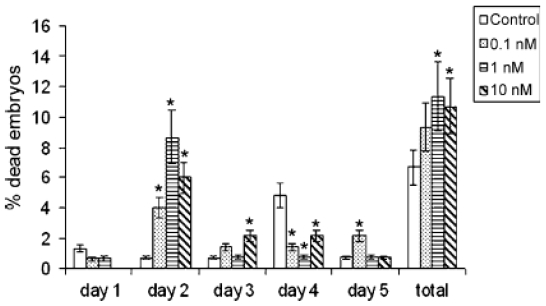
FETAX bioassay: time- and concentration-dependent effects of OA on embryo mortality (*p < 0.05 *versus* control). (Reprinted with permission from [80]).

**Figure 8:**
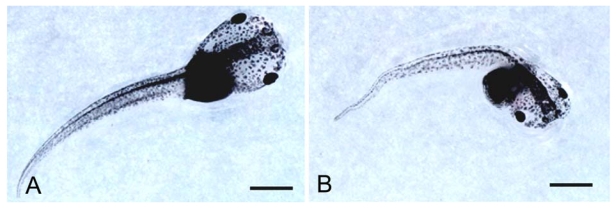
Images of control (A) and 1 nM OA treated (B) *X. laevis* early larval stages: note the tail folding and the reduced size at the end of toxin treatment. Bar = 1 mm. (Reprinted with permission from [80]).

*X. laevis* embryos at early gastrula stage (stage 10 [83]) were treated with different concentration of OA (0.1, 1 and 10 nM) for five days. Deceased and malformed embryos were counted daily, and at the end of the test the percentage of treated embryos showing delayed growth was evaluated by measuring head-tail length [80]. The morpho-functional modifications of surviving young larvae (stage 47) were also studied. The bioassay showed that OA affects the above parameters in a dose-correlated manner (Figure 7, [80]). Morphological observations of treated samples revealed a marked folding of the tail (Figure 8) that caused severe histological and histochemical damage to the nervous system (the most sensitive tissue) and to the tail skeletal musculature, while modifications also involved the intestine, liver and kidney. Indeed the rhombencephalon and spinal cord appeared reduced in size with irregularly arranged neuron cell bodies. The apoptotic figures counted in the rhombencephalon significantly increased compared to controls. Smaller, fewer and irregularly distributed muscle fibers, sometimes detaching at the periphery, were found in tail skeletal musculature. Depending on the extent of tail folding, there was a compression and/or reduction of the abdominal cavity with altered macroscopic and microscopic arrangements of the inner organs [80].

**Figure 9:**
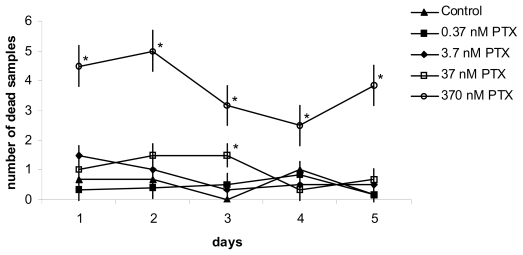
FETAX bioassay: time- and concentration- dependent effects of PTX on sample mortality (*p < 0.05 *versus* control). (Reprinted with permission from [81]).

**Figure 10:**
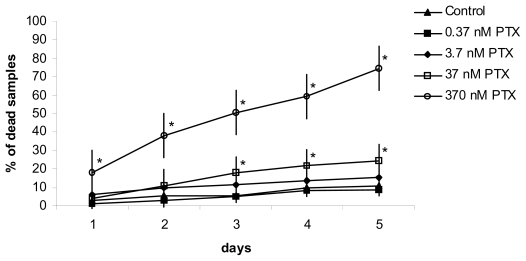
FETAX bioassay: percentage of dead samples compared to the initial embryo number (*p < 0.05 *versus* control). (Reprinted with permission from [81])

The toxicological effects of PTX evaluated by FETAX assay revealed evident impacts on embryo mortality, teratogenesis and growth at two toxin concentrations (370 and 37 nM) [81]. Significant mortality rates (Figure 9), peaks in malformed embryo number and delays in growth were found, and the initial sample population fell by about 80% at the end of the assay when using the higher dose (Figure 10).

The histological analysis of the surviving young larvae (stage 47) revealed structural changes compared with controls in the nervous and muscle tissue, even if some specimens did not show any significant histopathological modifications. A general reduction in the size of the main inner visceral organs (*i.e.*, intestine, pancreas and liver) was seen, but no morphological changes. Severe injury to the heart structure was observed in some specimens. No inflammatory response was observed [81].

On the basis of FETAX and histological results, molecular biology based experiments were also performed to assess more precisely the target of OA and PTX [82]. In particular, four genes involved in the early events of *X. laevis* development (specifically, neural and muscular specification and patterning), *i.e.*, *siamois*, *engrailed-2*, *bone morphogenetic protein 4* (*bmp4*), and *myogenic factor 5* (*myf5*) [84–90], were tested.

**Figure 11:**
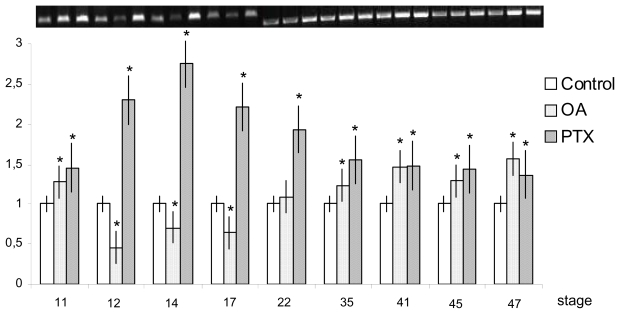
*bmp4* expression levels in *X. laevis* embryonic and early larval stages after toxin treatments. Bars represent standard deviation (SD) from the mean value of light intensity registered for each sample and normalized *versus* control value (*p < 0.05). (Reprinted with permission from [82]).

**Figure 12:**
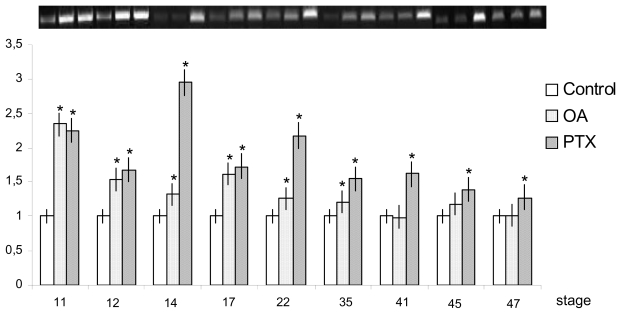
*myf5* expression levels in *X. laevis* embryonic and early larval stages after toxin treatments. Bars represent SD from the mean value of light intensity registered for each sample and normalized *versus* control value (*p < 0.05). (Reprinted with permission from [82]).

*X. laevis* embryos at early gastrula (stage 10 [83]) were treated with 10 nM OA [80] or 370 nM PTX, [81] and RT-PCR analyses were performed at the following stages of embryonic and post-embryonic development after different toxin exposure times: about 3 h (stage 11), 5 h (stage 12), 8 h (stage 14), 10 h (stage 17), 1 d (stage 26), 2 d (stage 35), 3 d (stage 41), 4 d (stage 45), 5 d (stage 47). Both toxins induced an over-expression of *siamois* and *engrailed-2. Siamois* was only found in the tested gastrulation stages (11 and 12). *Engrailed-2* expression levels were first detected at stages 14, increased at stage 26 and did not differ significantly from the controls at further development (stages 35–47). Different behavior was seen for *bmp4* and *myf5* (Figures 11, 12). Indeed, OA provoked a significant increase in *bmp4* in the earliest stage examined, a down-regulation from stages 12–17, and a renewed increase from the beginning of hatching onwards (stages 35–47). In contrast, *myf5* was up-regulated in all stages up to 35. PTX induced an over-expression of both *bmp4* and *myf5* during the embryonic and early larval development stages.

The results show that PTX induces an increase in expression levels in all tested genes, while the response to OA seems to be more stage-dependent, with the embryonic development stages more sensitive to the toxin than the larval stages [82]. More importantly, even though these processes deserve further studies, both OA and PTX seems able to interfere with gene expression, as it has recently been proposed also for YTX [26].

## 4. Conclusions

The general label of marine algal toxins combines very diverse molecules presenting different targets and specific modes of action. These toxins are currently studied in numerous models and reports of conflicting data are therefore not surprising. In this sense, the experiments performed in mussels are a good example of different outcomes dependent on experimental and animal conditions. Several researchers have concentrated their attention on the molecular structure, targets and toxicological properties of marine toxins. From our and other laboratory findings, the cytoskeleton, a structure finely integrated to cell functions, appears to be particularly susceptible to the mechanisms induced by the different toxins. However, the large number of organs and functions targeted by toxins such as YTX, OA and PTX should also prompt the adoption of new protocols, e.g., FETAX assay and molecular biology-based strategies, in order to study the influence that these toxins may exert on widely complex biological processes including, for instance, development.
